# Factors affecting the number of bacteria in saliva and oral care methods for the recovery of bacteria in contaminated saliva after brushing: a randomized controlled trial

**DOI:** 10.1186/s12903-023-03676-7

**Published:** 2023-11-24

**Authors:** Madoka Funahara, Runa Yamaguchi, Hiromi Honda, Misaki Matsuo, Wataru Fujii, Atsuko Nakamichi

**Affiliations:** School of Oral Health Sciences Faculty of Dentistry, Kyushu Dental University, 2-6-1 Manazuru, Kokura-Kita, Kitakyushu, Fukuoka 803-8580 Japan

**Keywords:** Brushing, Oral care, Bacterial counts, Mouthwash

## Abstract

**Background:**

Oral care is important in preventing aspiration pneumonia in older adults. However, it is not clear what kind of oral care can reduce the number of bacteria in saliva. The purposes of this study are to clarify whether there is a relationship between plaque amounts and salivary bacterial counts, and how bacteria dispersed into the oral cavity by brushing can be reduced.

**Methods:**

First, saliva samples were collected from 10 healthy adult volunteers after 30 h of unbrushing and after thorough brushing, and the total bacterial count was determined by real-time PCR. Next, 40 older adults attending an outpatient dental clinic were randomly assigned into two groups: a wiping group (20 patients) and a mouthwashing group (20 patients). Saliva was collected before and after brushing, and after wiping in the wiping group and after mouthwashing in the mouthwashing group, and the total bacterial count was quantified by real-time PCR.

**Results:**

In a study of volunteers, there was no association between plaque amounts and salivary bacterial counts. In a study of older adult patients, salivary bacterial counts were significantly higher in patients with higher oral hygiene index and fewer remaining teeth. Brushing increased salivary bacterial counts. Wiping did not significantly reduce the number of bacteria, while mouthwash returned the increased number of bacteria after brushing to the pre-brushing level.

**Conclusions:**

There is no direct relationship between the amount of plaque and the number of bacteria in saliva. Brushing disperses bacteria into the oral cavity, resulting in a marked increase in the number of bacteria in saliva. Wiping does not collect the dispersed bacteria, and it seems essential to rinse the mouth after brushing.

**Trial registration:**

UMIN000045854.

## Background

According to the Ministry of Health, Labour, and Welfare's 2021 Vital Statistics, pneumonia is the fifth leading cause of death among Japanese people, followed by aspiration pneumonia in sixth place [1], and the number of patients is expected to continue to increase in Japan's super-aged society. Aspiration pneumonia also significantly impairs the quality of life of patients, whose physical strength is declining due to symptoms such as fever, respiratory distress, and severe coughing. Therefore, aspiration pneumonia in older adults continues to be a challenge that requires a focus on prevention.

Aspiration pneumonia is a serious problem not only in older adults but also in perioperative patients undergoing highly invasive surgery [2]. Ventilator-associated pneumonia in patients receiving ventilator management in the intensive care unit after surgery is thought to be caused by the aspiration of oral bacteria such as *Staphylococcus aureus, Streptococcus pneumoniae*, and Gram-negative rods [3–6]. Also, after extubation of the tracheal tube, the risk of postoperative aspiration pneumonia may increase due to temporary dysphagia and the poor general conditions caused by surgical invasion. The risk of postoperative aspiration pneumonia may be higher after extubation of the tracheal tube owing to temporary dysphagia and a decreased general condition caused by surgical invasion. The main causes of aspiration pneumonia are thought to be decreased systemic resistance, impaired swallowing function, and higher influx of pathogenic microorganisms present in the oral cavity and pharynx [7–9]. In the field of dentistry, many attempts have been made to prevent the onset of aspiration pneumonia by providing rehabilitation to improve the swallowing function [10–12], and it is important to focus on this issue along with oral hygiene management. However, for older adult patients who require nursing care and those with intubated tracheal tubes, it is necessary to adopt an oral hygiene management approach. In this study, we focused on reducing the number of bacteria in the saliva by using an oral hygiene approach to prevent aspiration pneumonia. Oral hygiene management for the prevention of aspiration pneumonia has been widely studied, and brushing to remove dental plaque is the primary technique used to treat edentulous jaws in both older adults and perioperative patients [13]. However, it is unclear whether the removal of dental plaque reduces the number of bacteria in the saliva. A toothbrush brushes off the plaque adhering to the teeth, causing the plaque to mix with saliva. As a result, the number of bacteria in the saliva may increase immediately after brushing. This temporary increase in the bacteria in the saliva after brushing is not a major problem for healthy people because they can wash away the saliva mixed with dental plaque by rinsing their mouths immediately after brushing and can normally swallow the saliva mixed with the remaining bacteria. However, when brushing older adult patients requiring nursing care or those on ventilators, the collection of bacteria during and after brushing is important. The primary methods used to collect contaminated saliva from the oral cavity of these patients during brushing include wiping with a sponge brush or washing with water. Thus, we considered necessary to clarify the changes in the number of bacteria in the saliva immediately after brushing and the effect of these oral care techniques on salivary bacteria. Accordingly, the objectives of this study were to determine 1) the factors that affect salivary bacterial counts, 2) whether there is a relationship between the amount of dental plaque and salivary bacterial counts, and 3) how bacteria dispersed in the oral cavity by brushing can be recovered.

## Methods

### Differences in the number of bacteria in saliva depending on the degree of plaque adhesion

#### Study design

The purpose of this preliminary observational study was to evaluate the association of dental plaque with the number of bacteria in saliva. Saliva samples were collected from healthy adult volunteers with and without plaque for the same participants, and salivary bacterial counts were determined.

#### Ethical approval

This study was conducted in July 2020 in the Department of Oral Health Sciences, School of Dentistry, Kyushu Dental University, Kitakyushu, Japan. All procedures and materials were approved by the Ethics Committee of Kyushu Dental University (No. 20–38).

#### Participants

Eligibility criteria were adults who agreed to participate in the study, and exclusion criteria were those who were unable to mouthwash. Ten healthy female volunteers were included in this study, with a date of first registration of 7/28/2020.

#### Data collection procedures

##### Conduct of the study

The following factors were examined. Sex, age, number of remaining teeth, Oral Hygiene Index-Debris Index (OHI-DI) [14], plaque volume, Tongue Coating Index (TCI) [15], smoking and drinking status, hypertension, diabetes, and xerostomia were measured, and saliva was collected to determine the number of bacteria in it.

The TCI score was used to determine the degree of tongue coating via visual inspection. The tongue surface was divided into nine areas, and each area was rated on a 3-point scale (score 0–2). The total score was calculated by summing scores of the nine areas. The degree of oral dryness was measured by placing the surface of a dental mirror on the buccal mucosa, and when the buccal mucosa stuck to the mirror, it was considered dry. The number of bacteria in the saliva was measured using real-time PCR to determine the amount of bacterial DNA in the collected saliva.

Bacterial counts were measured in two saliva samples: one at the time of plaque adhesion and one after brushing. Saliva samples were collected and left unbrushed for 30 h to quantify the number of bacteria. Then, the OHI-DI scores were measured. Subsequently, the participants were instructed to brush their teeth for 3 min. After brushing, they were instructed to rinse their mouths with 180 mL of tap water. Three hours later, the specimens were collected, the number of bacteria in the saliva was determined, and the OHI-DI was measured using the same method as before brushing.

##### Real-time PCR

The number of oral microorganisms in saliva was measured as follows: First, participants were asked to spit approximately 1 ml of saliva into a bottle. Next, a filter paper was dipped into the bottle for 5 s to absorb the saliva. Collection was performed three times: before brushing, after brushing, and after wiping or rinsing the mouth. After saliva collection, the filter paper was placed in 500 µL PBS and allowed to stand for 30 min before DNA was collected from the samples. Real-time PCR was performed on the recovered DNA using specific primers, and the total bacterial count (Total Bacteria) was calculated from a standard curve created using synthetic DNA [16]. Targeted gene was 16S rRNA, and primer used in the study was as follows: forward: 5’-TCCTACGGGAGGCAGCAGT-3’, reverse: 5’-GGACTACCAGGGTATCTAATCCTGTT-3’). Real-time PCR was performed under the following conditions: thermal denaturation at 95 °C for 20 s, annealing at 62 °C for 90 s, and 40 cycles of DNA amplification. After amplification, fluorescent signals were detected at 95 °C: 15 s, 60 °C: 30 s, and 95 °C: 15 s. A melting curve was constructed to confirm the specificity of the amplified product.

#### Statistical analysis

Statistical analyses were performed using IBM SPSS Statistics Ver 23.0 (IBM, Tokyo, Japan). Corresponding t-tests were used to compare the number of bacteria in saliva at the time of plaque adhesion and at the time of plaque removal.

### Differences in the number of bacteria in saliva according to oral care methods

#### Study design

This single-center, randomized, controlled clinical trial was conducted at the Department of Oral Health Care, Kyushu Dental University Hospital (Kitakyushu, Japan) between October 2021 and December 2022. The main objective was to evaluate oral care methods using the number of bacteria in the saliva as an indicator, and the secondary objective was to evaluate the factors affecting the number of bacteria in the saliva.

#### Ethical approval

All procedures and materials were approved by the Ethics Committee of Kyushu Dental University (No. 21–25). All participants agreed to participate in the study and signed informed consent forms. This manuscript was prepared according to the CONSORT guidelines and registered on the University Hospital Medical Information Network study registration platform in Japan (UMIN000045854/ Registered date: 26/10/2021).

#### Participants

Eligibility criteria were adults who agreed to participate in the study, cleaned their mouths by themselves daily, and were capable of regular food intake, and exclusion criteria were those who were unable to mouthwash. Forty patients who visited the outpatient clinic of a university hospital for dental treatment were enrolled in the study with a date of first registration of 26/11/2021.

#### Data collection procedures

##### Conduct of the study

The participants were randomly divided into two groups by a simple randomization using the envelope method in a 1:1 ratio by one of the authors (MF): wiping and mouthwashing groups.

Before brushing, medical interviews and oral examinations were conducted, and TCI, xerostomia, and pre-brushing saliva samples were collected. Saliva samples were collected by placing a filter paper under the tongue of the participant for 5–10 s to absorb the saliva accumulated in the oral cavity and moistened at least 10 mm from the tip of the filter paper. The patients were brushed by two trained dental hygienists. The toothbrush used had a 10 mm wide × 20 mm head, normal bristle hardness, a flat-cut brushing surface, and a straight grip (STRIX DESIGN, Inc., Tokyo, Japan). The sponge brush used for cleaning was bale-shaped with a cut-like, uneven treatment on the scrubbing surface, 18 mm in diameter, 18 mm in length, and 150 mm in total length including the handle. The scrubbing method was used for brushing. The dental hygienist first brushed the participant's upper jaw from the buccal molars to the anterior teeth, then the palatal side of the maxilla, and finally the mandible in the same order for approximately 3 min.

After brushing, the participants were instructed to leave saliva in the oral cavity without swallowing or spitting it out and not to rinse their mouths. A second saliva sample was collected in the manner described above. Subsequently, according to the assigned oral care technique group, the wiping group wiped the buccal and lingual (palatal) mucosal surfaces of the dentition for approximately 2 min using a sponge brush. Two paper cups filled with water were used for wiping. The sponge brush that had been used to wipe the oral cavity was rinsed with one cup, and the other was used to rinse the sponge brush again, adjusting the water content. In the mouthwashing group, the participants were instructed to swish around the mouth with up to one paper cup of tap water (about 180 mL), so that the water was distributed throughout the mouth and on the right and left buccal sides. They then spit out the mouthwash solution. A third sample was collected after wiping or washing the mouth. Bacterial counts in the saliva were determined using real-time PCR, as described above.

#### Statistical analysis

Statistical analyses were performed using IBM SPSS Statistics Ver 23.0 (IBM, Tokyo, Japan). Corresponding t-tests were used to compare the number of bacteria in saliva at the time of plaque adhesion and at the time of plaque removal. Statistical analyses of bacterial counts before brushing, after brushing, and after wiping or rinsing were performed using the paired-samples t-test, followed by multiple comparisons using the Holm–Bonferroni method. Patient factors affecting the Total Bacterial count before brushing were examined using the Mann–Whitney U test, Spearman's rank correlation coefficient, and multiple regression analysis.

## Results

### Differences in the number of bacteria in saliva depending on the degree of plaque adhesion

**Fig. 1:**
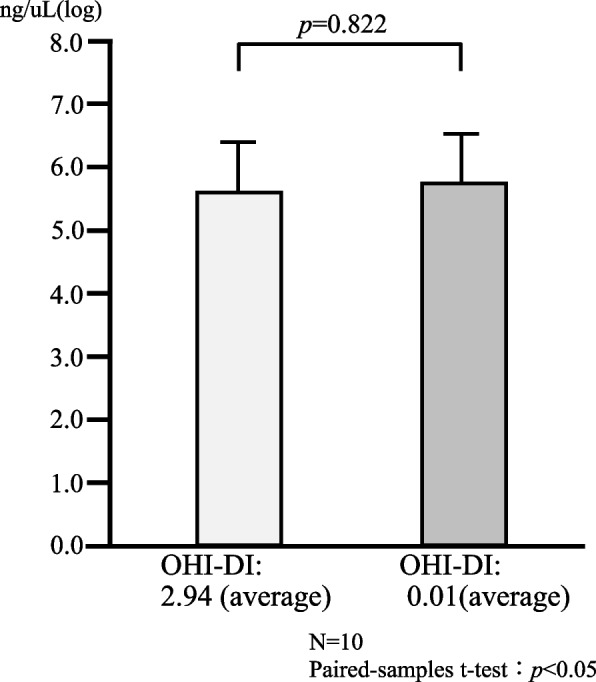
Relationship between OHI-DI score and salivary bacterial count. OHI-DI: Oral Hygiene Index—Debris Index

The number of bacteria in saliva was 5.80 ± 1.08 when the OHI-DI was 2.94 ± 0.39 and there was a large amount of plaque on the teeth, whereas the number of bacteria in saliva was 5.90 ± 1.35 when the OHI-DI was 0.012 ± 0.036 and there was almost no plaque (Fig. 1), showing no difference between the two groups. These results indicate that the number of bacteria in the saliva is not directly related to the number of bacteria in dental plaque.

### Differences in the number of bacteria in saliva depending on oral care methods

**Fig. 2:**
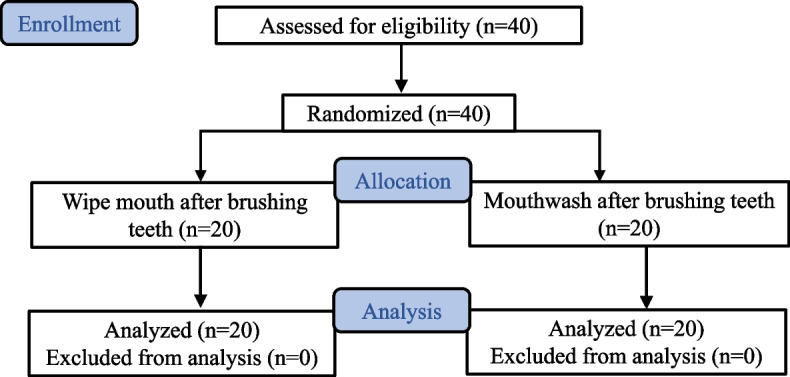
CONSORT flow diagram

The forty participants of the study were randomly divided into two groups (Fig. 2). The patients underwent maintenance and periodontal and prosthetic treatments. There were 17 males and 23 females, with a mean age of 70.8 years. The mean number of remaining teeth was 20.5, mean OHI-DI score was 1.42, and mean TCI score was 5.13 (Table 1). There were no differences in background factors between the two groups (wiping and mouthwashing).

**Table 1:** Participant characteristics

Variable		Number of participants / mean ± SD
Sex	male	17
	female	23
Age		70.83 ± 14.09
Number of teeth		20.50 ± 7.07
OHI-DI		1.42 ± 1.17
TCI		5.13 ± 3.78
Smoking	(-)	38
	( +)	2
Drinking	(-)	31
	( +)	9
High blood pressure	(-)	17
	( +)	23
Diabetes	(-)	38
	( +)	2
Dry mouth	(-)	23
	( +)	17

**Fig. 3:**
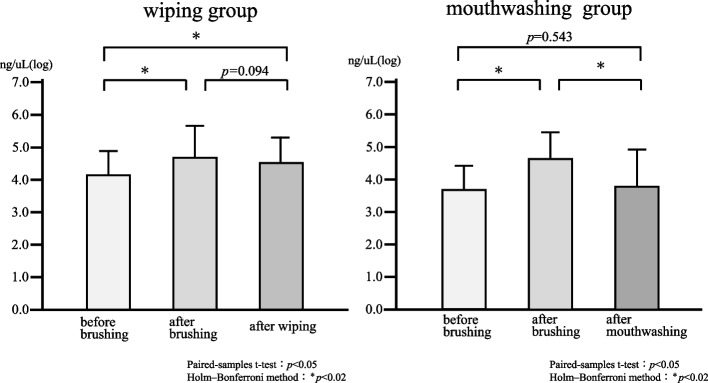
Total bacterial count before and after tooth brushing, and after oral care

First, factors affecting the number of bacteria in the saliva before brushing were examined in all 40 patients. Univariate analysis results showed that those with fewer remaining teeth (*p* = 0.002) and higher OHI-DI scores (*p* < 0.001) had significantly higher salivary bacterial counts (Table 2). Results from the multivariate analysis, in which items that were significant in the univariate analysis were entered as covariates, revealed that the number of remaining teeth (*p* = 0.042) and OHI-DI score (*p* = 0.018) were significant factors for an increase in the number of bacteria in the saliva (Table 3). Next, we examined the changes in salivary bacterial counts after oral care. There was no significant difference in the Total Bacterial count before brushing between the mouthwashing and wiping groups. Comparing the number of bacteria in saliva before and after brushing, there was a significant increase in the number of bacteria after brushing in both the mouthwashing and wiping groups. In the wiping group, the number of bacteria in the saliva after brushing and after wiping showed no significant difference (*p* = 0.094). The number of bacteria before brushing significantly increased than that after wiping (*p* < 0.001). In the mouthwashing group, the number of bacteria in the saliva after mouthwashing significantly decreased than that after brushing (*p* < 0.001). There was no significant difference between the number of bacteria before brushing and that after mouthwashing (*p* = 0.543) (Fig. 3).

**Table 2:** Factors related to the number of total bacteria in the saliva (univariate analysis)

i) Variable (categorical variable)		Median Logarithm of number of total bacteria (IQR)	*p-value* ^§^
Sex	male	4.0648 (1.02)	0.329
	female	4.0969 (1.52)	
Smoking	(-)	4.0809 (1.30)	0.831
	( +)	3.6564 (-)	
Drinking	(-)	4.1021 (1.31)	0.425
	( +)	3.7602 (1.31)	
High blood pressure	(-)	4.0648 (1.29)	0.705
	( +)	4.0969 (1.58)	
Diabetes	(-)	4.0635 (1.33)	0.503
	( +)	4.3759 (-)	
Dry mouth	(-)	4.0648 (1.52)	0.481
	( +)	4.0969 (1.25)	
ii) Variable (continuous variable)		Spearman's correlation coefficient	*p-value* ^†^
Age		0.262	0.102
Number of teeth		-0.478	^**^0.002
OHI-DI		0.531	^**^0.000
TCI		0.041	0.800

^§^Mann–Whitney U test

^†^Spearman's rank correlation coefficient

^*^p < 0.0

^**^p < 0.01

**Table 3:** Factors related to the number of total bacteria in the saliva (multivariate analysis)

Variable	Unstandardized coefficients		Standardized coefficients	95% confidence interval		*p*-value
	B	SE	β	lower	Upper	
Number of teeth	-0.039	0.019	-0.314	-0.077	-0.002	^*^0.042
OHI-DI	0.28	0.113	0.371	0.051	0.508	^*^0.018
TCI	0.025	0.032	0.107	-0.04	0.09	0.445

Multiple regression analysis

^*^p < 0.05

## Discussion

This study showed that bacteria that spread into the oral cavity by brushing cannot be completely recovered by wiping, and that mouthwashing is essential after brushing. In addition, although the amount of dental plaque does not directly affect the number of bacteria in saliva, it has been suggested that the number of bacteria in saliva may increase when the number of remaining teeth decreases, owing to poor oral hygiene, resulting in a decline in oral function.

Dental plaque is an aggregate of microorganisms and a risk factor for dental caries and periodontal diseases. Therefore, daily brushing of teeth is common to remove dental plaque. However, the number of bacteria in saliva contaminated by brushed-off dental plaque has not been examined. In this study, the number of Total Bacteria in the saliva increased immediately after brushing. This was thought to be a temporary increase because plaque adhering to the teeth was removed by brushing and mixed with saliva. Brushing by caregivers and medical personnel is typically performed for older adults and postoperative patients who are unable to brush by themselves. However, this increase in the number of bacteria in saliva immediately after brushing may increase the risk of aspiration pneumonia due to the aspiration of saliva in patients with impaired swallowing function. Therefore, it is necessary to be especially conscious of the need to collect contaminated saliva frequently during brushing and to establish a brushing method that prevents plaque from falling into the oral cavity during brushing.

The effectiveness of wiping and mouthwashing as a method for collecting contaminated saliva, which temporarily increases bacterial counts due to brushing, was investigated. In clinical practice, it is difficult for older adults requiring nursing care or for patients to rinse their mouths immediately after surgery, and caregivers often brush them with suction or wipes. However, the results of the present study indicated that wiping is insufficient for collecting bacteria during brushing. Hayashida et al. examined oral care methods for intubated patients and reported that washing with tap water effectively reduced the number of bacteria in pharyngeal effluents [17].

As described above, it is important to swish around the mouth or perform oral irrigation with water after brushing; however, when this is not possible, it is necessary to establish new oral care methods, such as wiping and mouthwashing with antiseptic agents. This study also examined the factors affecting the number of bacteria in the saliva before brushing. The results showed that the bacterial count was significantly higher when the number of remaining teeth was lower or the OHI-DI score was higher. Previous reports have identified age and xerostomia as factors affecting the number of bacteria in the saliva of perioperative patients [18]. Other studies have reported that factors such as the presence of food residue, xerostomia, inability to keep the mouth open, inability to mouthwash, and use of dentures affect the number of bacteria in the saliva of older adults in long-term care nursing homes [19]. Patients with high salivary bacterial counts in the present study had high OHI-DI scores but fewer remaining teeth, and the amount of dental plaque was not high when considered on an oral unit basis. In fact, when the number of bacteria in the saliva was measured in the same participant with a large amount of plaque and after the plaque was removed, no difference was observed. This suggests that plaque itself is not the source of salivary bacteria and that patients with high OHI-DI scores may have had poor oral hygiene over a long period of time, resulting in caries, periodontal disease and fewer remaining teeth. Tashiro et al. reported that a decrease in the number of remaining functioning teeth leads to a decrease in tongue pressure [20], and we previously reported that a decrease in tongue pressure leads to an increase in the number of bacteria in the saliva [21]. Poor oral hygiene may lead to a decrease in oral function, which in turn may increase the number of bacteria in the saliva. However, this study did not examine factors related to oral function, such as tongue pressure or masticatory force, which is an issue for future research.

This study has several limitations. First, the study was conducted on a small number of patients at a single institution, and it is not clear whether the results obtained can be generalized. In addition, oral function tests, which are thought to be the most important factors influencing salivary bacterial counts, were not performed. Furthermore, mouthwashes after brushing were performed with water, and the effect of mouthwashes with disinfectants, such as povidone-iodine, was not examined. We would like to investigate these points further in the future.

## Conclusions

The total number of bacteria in the saliva increased significantly after brushing compared to before brushing. Mouthwashing was an effective method of collecting contaminated saliva after brushing. Poor oral hygiene and a decrease in the number of remaining teeth were identified as factors associated with an increase in the total number of bacteria in saliva before brushing; however, the amount of plaque did not directly affect the number of bacteria in saliva.

## Data Availability

The datasets used and/or analyzed in this study are available from the corresponding author upon reasonable request.

## Abbreviations

OHI-DI: Oral Hygiene Index-Debris Index
TCI: Tongue coating index
SD: Standard deviation
